# Play and Supportive Environments Among Older Adults in the Context of Swedish Municipal Care: Protocol for the Development and Psychometric Evaluation of an Instrument to Measure Playfulness

**DOI:** 10.2196/86373

**Published:** 2026-04-13

**Authors:** Albert Westergren, Anna Bergman, Sofi Knaust Lidén, Catarina Nahlén Bose, Anna Nivestam, Annika Norell, Petra Nilsson Lindström, Maria Haak

**Affiliations:** 1 Faculty of Health Sciences, Kristianstad University, SE-291 88, Kristianstad, Sweden, +46 44 250 38 41; 2 Department of Health Sciences, Swedish Red Cross University, Stockholm, Sweden; 3 School of Behavioural, Social and Legal Sciences, Örebro University, Örebro, Sweden

**Keywords:** concept development, existential philosophy, instrument development, literature review, mixed methods, municipal care, older adults, person-centered care, playfulness, psychometrics, lifeworld theory

## Abstract

**Background:**

Playfulness—being and acting playful—is often associated with childhood, yet evidence suggests that it remains a meaningful resource throughout life. In later life, playfulness may support social connectedness, emotional well-being, and a sense of agency, even in contexts of illness or institutional living. Playfulness encompasses not only observable playful activities but also an inner disposition, such as curiosity, humor, or spontaneity, which may be constrained by environmental barriers, aging, or functional limitations. Despite its potential relevance for health and person-centered care, playfulness remains underexplored in gerontological and caregiving research. No validated instrument currently exists to assess playfulness among older adults in Swedish municipal care. This research program addresses this gap by clarifying the concepts of play and playfulness and by developing and psychometrically evaluating a new instrument, Play and Supportive Environments (PLAY-SE).

**Objective:**

The overall aim of the program is to clarify and operationalize the interrelated concepts of playfulness and playful activities among older adults receiving municipal care and to develop an instrument suitable for psychometric testing.

**Methods:**

The program applies a hybrid model of concept development combined with an exploratory sequential mixed methods design. Phase 1 involves literature reviews and qualitative studies with older adults and staff to explore and define the lived meanings of playfulness. These findings inform item generation and refinement of the PLAY-SE instrument. Phase 2 includes content validation, cognitive interviews, pilot testing, and large-scale psychometric evaluation using both classical test theory and Rasch measurement theory.

**Results:**

Two PhD students were recruited to the program in September 2024 and September 2025, and an expert group was established in autumn 2025. The PhD students are funded, for four years each, by Kristianstad University (from 2024) and Red Cross University College (from 2025). Ethical approval for the qualitative studies in phase 1 was granted by the Swedish Ethical Review Authority (2025-00211-01; decision date: February 3, 2025). Data collection for qualitative interviews with older adults in municipal care was conducted between February and April 2025. Fifteen older adults (aged 68‐100 y) were interviewed in nursing home settings. The phenomenological findings from phase 1.1 have been published in March 2026. Additional qualitative interviews and focus groups with staff are scheduled for 2026 to 2027. Pilot testing of the first version of the PLAY-SE instrument is planned for autumn 2026, followed by large-scale psychometric validation between 2027 and 2029.

**Conclusions:**

This program establishes a structured and theoretically grounded process for developing and validating an instrument to assess playfulness in later life. By integrating qualitative exploration with modern psychometric approaches, the PLAY-SE instrument is intended to support future research and contribute to the development of person-centered practices in municipal elder care.

## Introduction

### Background

**Figure 1.**
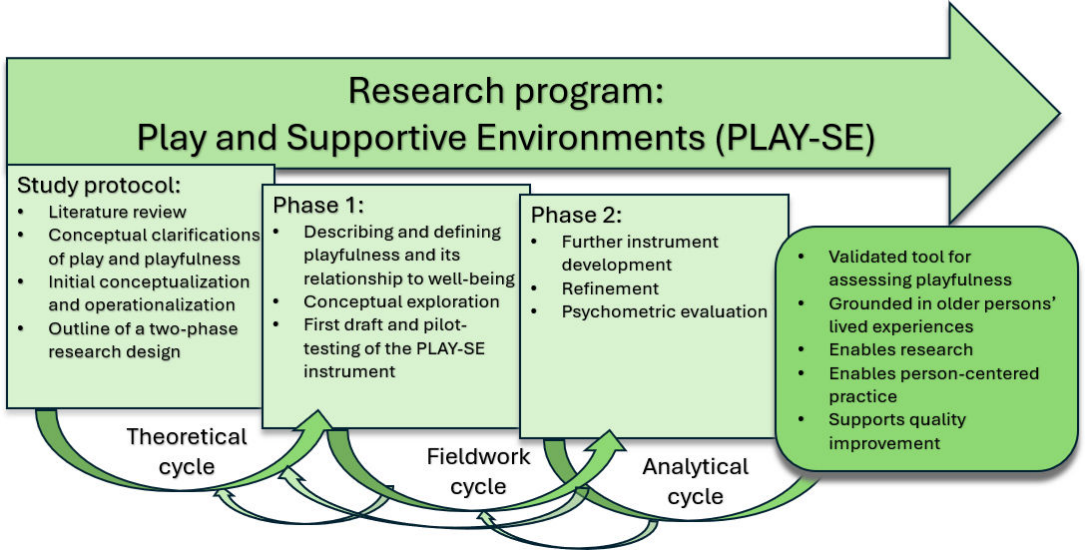
Overview of the Play and Supportive Environments program, the study protocol, and the 2-phase research design. Three cycles are applied iteratively to deepen both the conceptual understanding and the operationalization of playfulness.

Playfulness (ie, being and acting playful) is increasingly recognized as important for older adults’ health, well-being, and dignity, even in contexts of illness or institutional care. Yet, research and practice still lack validated instruments that capture this phenomenon from the perspective of older adults themselves. In this study protocol, we describe a research program organized into 2 interrelated phases (Figure 1). The first phase explores the lived experience and meanings of playfulness in later life, while the second focuses on the development and psychometric evaluation of a new instrument (Play and Supportive Environments [PLAY-SE]). To facilitate this process, the protocol provides conceptual clarifications of playfulness and play, as well as an initial conceptualization of the domains to be included in the instrument.

Although being playful and acting playfully are often associated with childhood, studies show that playfulness remains a stable trait across the lifespan, with older adults not inherently less interested in playful engagement [1-3]. Nevertheless, dominant life-course frameworks have traditionally linked playfulness to childhood, socialization to adolescence, work to adulthood, and passive leisure to old age, which has contributed to limited research attention on playfulness in later life [4]. Expanding this view is important, as playfulness in later life has been linked to well-being, creativity, cognitive health, and social connectedness [4,5].

Being and acting playfully are closely tied to a person’s sense of identity, purpose, and relational connectedness [6], core components of person-centered care as articulated by McCormack and McCance [7]. Their framework emphasizes 4 modes of being: being with self, being in relation, being in a social world, and being in place. Engaging in playful activities or expressing a playful attitude may facilitate all these dimensions, thereby supporting the dignity, autonomy, and well-being of older adults in care. In contexts where daily routines are often shaped by institutional or structured living environments, playfulness can represent a moment of agency (ability to act independently, make choices) and self-expression [8,9].

Being and acting playful can also be understood through an existential lens. According to Merleau-Ponty [10], the body is the primary site of being in the world. In situations where mobility or independence is compromised, opportunities for unreflective participation in playful activities may diminish. Nonetheless, playfulness as an attitude or mode of being may remain accessible and vital, offering continuity and meaning even during times of change. This perspective reinforces the importance of enabling older adults to express their playfulness, even within the constraints of care environments.

In the context of municipal care, older adults living in long-term care facilities often report limited access to meaningful, self-chosen activities and social engagement. Both a Swedish report [11] and international studies [12,13] highlight widespread boredom, monotony, and passivity—conditions that stand in contrast to legal aims for an active and meaningful life in the community [14]. At the same time, international research suggests that playfulness may contribute to physical, emotional, and social well-being throughout the lifespan [15-17].

### Literature Review

A structured exploratory literature review was conducted in May 2025 using the research platform Elicit [18], which applies large-scale paper search and screening based on predefined inclusion criteria. The guiding research question was: “What perspectives do older adults (65 years or older) have on playfulness and play, and what measures have been developed with a special focus on playfulness and play for older adults? In addition, what dimensions of play and/or playfulness are captured by these measures?” The search strategy included both the terms *playfulness* and *play*, since playfulness is often described as a state characterized by elements of play [19].

The search was conducted within the Semantic Scholar corpus (>126 million academic papers) and retrieved the 50 most relevant records, which were screened according to population age, study focus, study design, and relevance. Ten studies were initially included from this screening process. In addition, 3 studies were identified through backward citation tracking. The final sample comprised qualitative interview and participatory design studies [8,9,20-22], quantitative survey-based investigations [2,3,23,24], instrument development studies [25-27], and 1 narrative review [28]. From these, 3 validated instruments assessing playfulness in older adults were identified: the Older Adult Playfulness Scale (OAP) [27], the Other-Directed, Light-Hearted, Intellectual, and Whimsical (OLIW) Playfulness Scale [23], and the Short Measure of Adult Playfulness (SMAP) [2]. The following sections summarize the findings from this review, beginning with perspectives on play and playfulness among older adults and continuing with validated measures used in this field.

Older adults describe play and playfulness as multidimensional constructs encompassing cognitive, emotional, social, and psychological aspects. These dimensions are associated with identity, continuity, and vitality in later life [2,8,20]. For example, in an interview study of older adults aged 62 to 89 (mean age 74 y), play was described as a source of enjoyment, structure, and personal meaning in everyday life [9].

Studies link playfulness to well-being, showing that it helps older adults reframe everyday challenges, maintain social ties, and support mental and physical health. In a study of persons aged 50 to 98 years, higher playfulness was associated with greater life satisfaction and flourishing [3]. Similarly, a survey study involving participants aged 60 years and older found that self-reported playfulness, especially in social contexts, was positively correlated with well-being [23].

Recent research has further strengthened this empirical foundation. For example, Brauer et al [3] demonstrated positive associations between playfulness and life satisfaction, character strengths, and flourishing in middle and older age, while Clifford et al [24], in a general adult sample, found that playfulness was linked to adaptive coping and reduced stress. Although not restricted to older adults, these findings reinforce the relevance of playfulness as a health-related resource across adulthood and into later life.

Qualitative studies also shed light on the significance of play. Burr et al [8], through in-depth interviews with older adults (aged 69‐88 y, mean age 81 y, SD 6.06 y), emphasized play’s role in shaping life narratives and creating meaning. Schutter and Abeele [20,21] used contextual inquiries and participatory design methods with participants aged 68 to 80 years and 50 to 72 years to explore how older adults perceive meaningful play, particularly in digital contexts. These studies found that play supports connectedness, nurtures the self and others, and enables meaningful societal engagement through shared experiences and intergenerational interaction.

Play in digital environments has also been explored through ethnographic and participatory design studies. Sayago et al [22] demonstrated that older adults (aged 60‐85 y) actively engage in cocreating digital play experiences, possibly challenging ageist assumptions. In addition, Loos [28], in a literature review, identified social interaction, usability, and enjoyment as key motivators for engaging with exergames and digital play platforms.

Across both qualitative and quantitative studies, the social aspect of playfulness, especially the “other-directed” facet (enjoyment of others’ company and using playfulness to create positive social interactions), is consistently highlighted. This includes using humor and lightheartedness to foster relationships and navigate social tensions [3,23]. Themes such as connectedness, continuity, and participation also emerged in qualitative narratives, highlighting play’s role in enhancing older adults’ sense of agency and well-being [9,20].

Three validated instruments were identified in the review as being used to assess playfulness in older adults:

- The OAP Scale, developed through mixed methods research and a classical test theory (CTT) approach [27], was designed specifically for older populations (exact age range not specified) and focuses on unique age-related expressions of playfulness. The OAP includes 15 items divided into 4 factors: psychologically upbeat, behaviorally impish, cognitively spontaneous, and amusing.
- The OLIW Playfulness Scale (used by [23]) assesses 4 key dimensions: other-directed, light-hearted, intellectual, and whimsical traits. The instrument was applied in a quantitative survey targeting older adults aged 60 years and older. The instrument was developed by Proyer [26] using a CTT approach. It focuses on attitudes and actions in relation to playfulness and contains 28 items divided into 4 traits (as described above).
- The SMAP [2] was validated in a cross-sectional study with participants aged 18 to 92 years (mean age 45), offering insight into the intensity, frequency, and ease of initiating playful behavior across the lifespan. The SMAP was developed by Proyer [25] using a CTT approach. It has 5 items: i1, I am a playful person; i2, Good friends would describe me as a playful person; i3, I frequently do playful things in my daily life; i4, It does not take much for me to change from a serious to a playful frame of mind; and i5, Sometimes, I completely forget about the time and am absorbed in playful activity.

Taken together, qualitative studies highlighting meaning, continuity, and connectedness [8,9,20,21] and quantitative research linking playfulness with well-being [2,3,23] suggest that playfulness in older age is complex, contextual, and relational. However, existing instruments primarily conceptualize playfulness as a personality trait and have been developed for general adult populations. They place less emphasis on how playfulness is enacted in everyday life within care contexts or on how environmental and functional constraints may shape this expression.

Valid and age-sensitive instruments, responsive to functional limitations and municipal care environments, are therefore needed to capture these nuances in both research and practice. PLAY-SE will be grounded in older adults’ lived experiences within Swedish municipal care and will integrate perspectives from caring science and person-centered care. It will be developed with explicit Rasch-based psychometric intentions to ensure appropriate targeting and invariant measurement in this specific population. For these reasons, adaptation of existing instruments was considered insufficient to meet the conceptual and methodological aims of this research program.

### Theoretical Underpinnings

This program will be conducted from a person-centered perspective, emphasizing the ethical aim of a good life, lived with and for others, within fair and equitable institutions [29]. To explore playfulness in older adults living in long-term care facilities, we apply the 4 modes of being a person: being with self, being in relation, being in a social world, and being in place, as described by McCormack and McCance [7]. In addition, existential philosophy provides a lens through which human beings are not seen as predetermined. Although factual limitations shape our lives, they do not ultimately define who we are. Instead, we are always self-making beings, responsible for the meanings we give through our choices, the totality of which makes us who we are [30]. In caring science, this tension between determinism and freedom is understood as the interplay of vulnerability and freedom [31].

To achieve research depth that embraces lived experiences as meanings, a phenomenological reflective lifeworld research (RLR) approach will be adopted (in the first phase of the program), as outlined by Dahlberg et al [32]. In this way, the program will explore older adults’ lived experiences of being playful while living in long-term care facilities. In RLR, the phenomenon is the central focus and is described through its essential meaning and constituents. The approach draws on the philosophical underpinnings of hermeneutics and phenomenology as described by Gadamer [33], Husserl [34], and Merleau-Ponty [10].

RLR provides methodological principles such as openness, flexibility, and bridling. Openness deals with the demand for sensitivity to the phenomenon being studied and entails having the capacity to be surprised and sensitive to the unpredictable and unexpected. Through this open attitude, the objects of the lifeworld may reveal themselves as different from how they were previously assumed to be [32]. To bridle means to openly return to the things themselves without making definitive what is indefinite, so as not to understand too quickly. In addition, within the bridling attitude, one moves beyond natural understanding and restrains one’s preunderstanding, including personal beliefs, theories, and other assumptions, so that they do not mislead the understanding of meaning.

### Conceptual Framework and Hierarchy

As part of the program, the PLAY-SE instrument will be developed at the end of phase 1 and refined and evaluated during phase 2 (Figure 1). The development of a psychometric instrument typically begins with qualitative conceptualization [35], where the construct of interest (eg, playfulness) is explored through interviews, observations, and/or literature reviews to ensure content is grounded in lived experience (phase 1). Based on these insights, developers generate domains and items that reflect different facets or levels of the construct, along with a response format suited to capture variation in intensity or frequency. To enable measurement on a hierarchical scale or “ruler,” items should ideally vary in difficulty or endorsement level, allowing for ordering along a unidimensional latent trait. In this way, the qualitative and theoretical work contributes to defining an outcome space [36], that is, a structured representation of the range and organization of possible expressions of playfulness, ranging from lower to higher levels, from being playful to acting playfully. This is followed by pilot testing and psychometric evaluation using approaches such as Rasch model analysis, which assesses item fit, scale targeting, and unidimensionality, thereby supporting the creation of an interval-level measure. Final validation typically includes analyses of reliability, validity, and responsiveness across diverse samples [35,36].

Psychometrics is the scientific study of measurement instruments, such as rating scales [37]. Traditional approaches, often referred to as CTT, rely on correlation-based techniques such as Cronbach α, principal component analysis, and factor analysis [37,38]. Although widely used, CTT assumes normally distributed, continuous data, does not ensure invariant measurement across persons or contexts, and treats summed scores as interval-level data, which limits generalizability and validity [39]. Rasch measurement theory (RMT), by contrast, offers a probabilistic framework within modern test theory [40]. By modeling the probability of endorsing an item based on person ability and item difficulty, Rasch model analysis enables unidimensional, invariant, and interval-level measurement. Its grounding in measurement principles comparable to the physical sciences allows for alignment between individual- and group-level structures, leading some to regard the Rasch model as the ideal standard for scientific measurement [41-43]. This approach enables quality-assured comparisons both within individuals over time and between individuals [44]. Consequently, RMT provides a foundation for truly person-centered research, focusing on measurement with and for the individual person.

### Conceptual Clarifications

In this program, a distinction is made between playful activities (sometimes referred to simply as “play”) and playfulness, while recognizing that the terms are often used interchangeably in everyday conversation. Theoretically, however, they represent 2 related yet distinct constructs.

- Play refers to activities undertaken for the sheer enjoyment of the experience—activities that feel fun, creative, or relaxing. Play can be solitary or social, such as crafting, playing games, joking, dancing, painting, imagining, or trying something new. Importantly, play is not driven by external goals or outcomes; its value lies in the experience and the meaning it holds for the person.
- Playfulness, on the other hand, describes a personal orientation or way of being playful, characterized by the ability to experience joy, express curiosity, use imagination, and find amusement in everyday situations. As a personality trait or state of mind, playfulness shapes how persons approach and interpret the world around them.

While playfulness can lead to playful activities, and playful activities may foster playfulness, they are not synonymous. In scientific research, it is valuable to distinguish between these concepts to avoid conceptual overlap and ensure analytical clarity. For instance, an older adult may retain a playful disposition (being playful) but be hindered from acting playfully due to situational and environmental circumstances, such as illness and institutional routines. In such circumstances, differentiating between inner orientation and outward expression becomes essential.

Moreover, “playful activities” may be understood as a broader construct than “play,” since it includes not only traditional forms of play (eg, games, hobbies, creative pursuits) but also everyday situations infused with humor, curiosity, or imagination. Recognizing these distinctions between being playful and acting playfully and between play and playful activities allows for a more nuanced understanding of how playfulness contributes to well-being in later life. This perspective also reflects prior research highlighting the multidimensional and contextual character of playfulness among older adults [2,3,8,19].

### Initial Conceptualization and Theoretical Hierarchy

**Figure 2.**
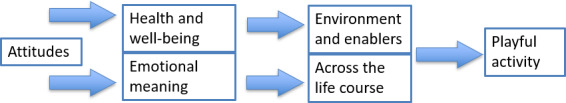
Hierarchical structure for the Play and Supportive Environments instrument: domains across increasing levels of playfulness.

As part of this review and study protocol, an initial conceptualization and theoretical hierarchy for the PLAY-SE instrument were developed. This process drew on insights from the literature, together with conceptual reasoning, providing a theoretically grounded basis for the forthcoming item generation and psychometric evaluation. The conceptualization and hierarchy will be further elaborated in forthcoming discussions within the research team, drawing on findings from phase 1 and serving as a springboard for phase 2 of the program. The PLAY-SE will be a self-administered instrument comprising approximately 25 statements across 6 preliminary conceptual domains: : “Across the Life Course,” “Attitudes,” “Environment and Enablers,” “Emotional Meaning,” “Health and Well-being,” and “Playful Activity.” While designed for independent completion, support from staff should be offered, if needed, to ensure that all participants can engage with the instrument on their own terms. Higher scores will reflect greater levels of playfulness or engagement in play among older adults receiving municipal care (Figure 2).

Each domain will reflect a distinct aspect of the phenomenon and contribute to a comprehensive understanding of how playfulness may be experienced and expressed in later life. These domains are not only conceptually defined but also hypothetically arranged along a continuum, ranging from abstract attitudes and internalized values to embodied, enacted expressions of playfulness through playful activities. This structure reflects the idea that playfulness can manifest at different levels of engagement, “from potential to practice” (as illustrated in Figure 2). Further to the left indicates that it is easiest to agree with a statement (a lower degree of playfulness is required). Further to the right indicates that it is hardest to agree with a statement (a higher degree of playfulness is required).

Compared to existing measures (OAP, OLIW, SMAP), the proposed domains extend beyond trait-level orientations by explicitly capturing both being playful and acting playfully, as well as environmental enablers and constraints. In this way, PLAY-SE aims to reflect the lived experience of older adults in municipal care more fully than previous instruments, thereby addressing important gaps in the assessment of playfulness in later life.

This structure reflects the idea that playfulness can manifest at different levels of engagement, “from potential to practice” (as further explained in Table 1).

**Table 1.** Domains, purpose, and arguments for hypothetical positions regarding playfulness and playful activity.

Domain	Purpose	Hypothetical position
1. Attitudes	Captures norms and self-perceptions related to play.	Considered the easiest domain to endorse, primarily involving the expression of general opinions (eg, “older adults can play”) without requiring personal engagement.
2. Health and well-being	Captures the perceived impact of play on health and well-being.	Generally easy to agree with; many older adults recognize the positive effects of play, even if they do not actively engage in it themselves.
3. Emotional meaning	Assesses how important play is and what it feels like to lose access to it.	Requires a more personal and emotional connection to the role of play, though it can still be understood hypothetically.
4. Environment and enablers	Captures how the environment and the body influence the possibility of engaging in play.	Reflects a willingness to play, acknowledging that expression depends on external conditions such as physical ability, institutional rules, or support.
5. Across the life course	Measures self-image and the continuity of playfulness throughout the lifespan.	Requires a stronger personal anchoring, as identifying oneself as playful over time implies a deeper integration of playfulness into one’s identity.
6. Playful activity	Captures behavioral expressions of play.	The most demanding domain reflects concrete engagement in playful actions and the creation of playful situations, indicating the highest level of expressed playfulness.

The 6 proposed domains for the PLAY-SE instrument are specifically designed to bridge older adults’ lived experiences with complex theoretical perspectives (such as person-centered care, lifeworld theory, and existential philosophy) by translating abstract concepts into dimensions that are both measurable and meaningful to the individual in their context. The domains collectively ensure that the instrument does the following:

- Centers the person and their subjective meanings (person-centeredness). Mainly captured by the domains of *attitudes* (toward play) and *emotional meaning*, which reflect personal values, emotional relevance, and how individuals perceive the role of play in their own lives.
- Reflects the totality of human experience—body, time, space, emotion, and sociality (lifeworld theory). Mainly captured by *environment and enablers*, *across the life course*, and *health and well-being,* which together address the spatial, temporal, emotional, physical, and social or intersubjective aspects of being playful in everyday life.
- Accounts for existential challenges and choices, such as freedom, limitation, loss, and identity (existential philosophy). Mainly captured by *playful activity*, *emotional meaning*, and *across the life course*, which reflect the tension between agency and constraint, the significance of loss, and the continuity of identity through play.

### Rationale and Aims

#### Overall Aim

The overall aim is to further clarify and operationalize the interrelated concepts of playfulness and playful activities among older adults receiving municipal care and to develop an instrument suitable for psychometric testing.

#### Phase 1—Conceptual Exploration

The overall aim of phase 1 is to deepen understanding of playfulness among older adults living in long-term care facilities. This includes exploring their lived experiences, engagement in playful activities, and the perspectives of care staff. A further objective is to examine how playfulness relates to well-being in this population.

Specific objectives are as follows:

1. To describe playfulness as experienced by older adults living in long-term care facilities
2. To explore older adults’ experiences of engaging in playful activities and their relation to health and well-being
3. To investigate activity coordinators’ and managers’ experiences of older adults’ possibilities and barriers to express their playfulness
4. To develop a first version of the PLAY-SE instrument, including initial item generation and pilot testing to explore its measurement properties and guide further refinement

#### Phase 2—Instrument Development and Psychometric Evaluation

The overall aim of phase 2 is to advance the development of the PLAY-SE instrument by exploring its theoretically developed domains against empirical data, refining its conceptual structure, and strengthening content validity [36]. This phase will also involve systematic psychometric evaluation, including assessments of usability, reliability, dimensionality, and validity, to ensure that the instrument provides a robust and nuanced measure of playfulness among older adults in the context of illness or institutional care.

Specific objectives are as follows:

1. To strengthen the theoretical and conceptual basis for the instrument
2. To assess content validity and usability through interviews
3. To conduct an initial psychometric evaluation
4. To validate a revised version using a larger, representative sample

This phase will also examine associations with the SMAP [25] and indicators of well-being among older adults receiving municipal care and service support.

## Methods

### Overall Design

Inspired by Huber et al [45] and the “hybrid model of concept development” [46], this program applies a hybrid model of concept development combined with an embedded mixed methods design. The hybrid model is particularly suited for refining and clarifying complex concepts by integrating theoretical insights with empirical observations. Concept development enhances conceptual clarity by identifying the essential attributes of a construct and translating them into measurable dimensions.

The hybrid model unfolds in 3 interrelated cycles (Figure 1):

1. Theoretical cycle: A literature review is conducted to examine how the concept has been defined and measured previously. In this protocol, this cycle has been initiated through a structured exploratory literature review using the Elicit platform and a synthesis of prior studies (see Literature Review). These findings inform conceptual clarifications of play and playfulness (see Conceptual Clarifications), tailored to the study’s aims.
2. Fieldwork cycle: Empirical data are collected and analyzed to investigate how the concept is expressed in real-world contexts. In phase 1, phenomenological interviews with older adults in municipal care provide meaning units that expand, refine, or challenge the preliminary theoretical understanding.
3. Analytical cycle: Empirical findings are reintegrated with the theoretical groundwork to develop a refined conceptual description. This refined description directly informs item generation and domain structure for the PLAY-SE instrument in phase 2. Thus, qualitative findings are systematically translated into candidate items, which are subsequently evaluated and refined through cognitive interviews and psychometric testing.

The cycles are applied iteratively, meaning that quantitative findings from pilot testing and Rasch analysis may prompt further conceptual reflection to ensure alignment between empirical performance and theoretical meaning. Together, these cycles underpin the 2 phases of the PLAY-SE program: phase 1 (conceptual exploration) and phase 2 (instrument development and psychometric evaluation).

### Setting and Participants

The program will be conducted in residential care facilities (ie, nursing homes) and in ordinary housing among persons receiving municipal care support in Sweden. Participants will include older adults aged 65 years and older, as well as activity coordinators and managers involved in municipal elder care.

Inclusion criteria (phase 1 and phase 2) were as follows:

- Older adults aged 65 years or older
- Receiving municipal care support, either in residential care facilities or in their own homes
- Able to provide informed consent
- Sufficient cognitive and communicative ability to participate in interviews, surveys, or focus groups

Exclusion criteria (phase 1 and phase 2) were as follows:

- Severe cognitive impairment or acute illness that prevents informed consent or meaningful participation
- Non-Swedish–speaking persons (due to the instrument being developed and tested in Swedish)

Preliminary sample size of phase 1 (conceptual exploration) was as follows:

- Approximately 15 to 25 older adults for qualitative interviews and 10 to 15 activity coordinators or managers for focus groups (guided by data saturation).

Preliminary sample size of phase 2 (instrument development and psychometric evaluation) was as follows:

- Content validity interviews with 10 to 15 older adults and 6 to 10 experts.
- A pilot survey including approximately 100 older adults to test the PLAY-SE instrument, version 1, and explore its initial measurement properties, as well as associations with SMAP [25] and well-being indicators.
- Large-scale validation study including 250 to 500 older adults to confirm psychometric properties (CTT and modern test theory).

### Sampling Strategy

A purposive sampling strategy will be used in phase 1 to ensure variation in gender, age, and relevant care contexts within municipal elder care. The aim is not statistical representativeness but conceptual variation to inform domain development, with particular emphasis on older adults living in residential care facilities.

For phase 2 (psychometric testing), a stratified sampling approach will be applied across multiple participating municipalities, including both urban and rural contexts. Recruitment will focus primarily on older adults receiving municipal care support in ordinary housing while ensuring sufficient variation in demographic and contextual characteristics. Eligible individuals will be invited consecutively or through structured random selection, where feasible. This approach aims to enhance representativeness within the intended target population while acknowledging practical constraints within municipal services.

To reduce recruitment bias, initial contact will be facilitated by designated care staff, but final eligibility confirmation and informed consent will be handled directly by the research team. Recruitment procedures will be documented to allow transparent reporting of participation rates and sample characteristics.

### Sample Size Considerations

The planned sample sizes are based on methodological considerations specific to qualitative research, CTT, and RMT.

For the qualitative interviews in phase 1 (approximately 15‐25 older adults), the sample size is guided by the principle of conceptual saturation and information power, where the aim is depth and variation rather than statistical representativeness [32]. Recruitment will continue until thematic depth and conceptual clarity are achieved across variations in gender, care setting, and functional status.

For pilot testing (approximately 100 participants), the sample size is considered sufficient for preliminary evaluation under CTT, including the assessment of internal consistency, item-total correlations, floor and ceiling effects, and exploratory dimensionality [37,38,47]. Samples of around 100 participants are commonly recommended for early-stage scale refinement to identify poorly functioning items and evaluate reliability coefficients such as Cronbach α [38].

For large-scale validation (250‐500 participants), the sample size is aligned with recommendations for RMT. Samples of approximately 250 participants allow reasonably stable estimation of item difficulty parameters, while samples approaching 500 participants improve the precision of item calibration and enable robust testing of differential item functioning (DIF) across subgroups [48-50]. Larger samples also strengthen the evaluation of unidimensionality and local independence and support more precise targeting of the instrument [51].

Together, these staged sample sizes are intended to ensure conceptual validity in phase 1 and rigorous psychometric evaluation in phase 2, in line with best practice recommendations for scale development.

### Recruitment Procedures

Participants will be recruited from both residential care facilities and ordinary housing with municipal home care support. Recruitment procedures will be adapted to each setting while maintaining consistent inclusion criteria. In residential care, responsible nurses or care managers will identify potentially eligible participants based on documented eligibility criteria and provide initial study information. In home care settings, recruitment will occur through designated contact persons within municipal services who distribute study information to eligible individuals. In all settings, final eligibility and informed consent will be confirmed directly by the research team to ensure voluntariness and comprehension.

### Assessment of Cognitive Ability

Eligibility requires sufficient cognitive and communicative capacity to understand study information and provide informed consent. Formal cognitive screening instruments will not be administered as part of the study. Instead, the assessment of cognitive capacity will be based on structured clinical judgment conducted in collaboration with responsible nurses or care staff, drawing on documented cognitive status in medical records and their ongoing knowledge of the individual’s functioning.

The assessment will focus on the participant’s ability to do the following:

1. Understand the purpose of the study
2. Comprehend what participation entails
3. Express a voluntary decision regarding participation

If uncertainty arises regarding decisional capacity, the individual will not be included. The basis for inclusion decisions will be documented to ensure procedural consistency.

### Administration Procedures and Mode Effects

The PLAY-SE instrument is designed for self-administration. However, given the target population, some participants may require assistance (eg, due to visual impairment or motor limitations). To ensure procedural consistency, assistance will follow a standardized protocol. Support will be limited to reading items aloud and recording responses as expressed by the participant, without interpretation, rephrasing, or prompting. The mode of administration (independent completion vs assisted completion) will be documented for each participant. This variable will be included in subsequent psychometric analyses.

Potential mode effects will be examined analytically. In the Rasch analysis, DIF will be tested across administration modes to determine whether assisted completion systematically influences item responses. If significant DIF is identified, its impact on measurement invariance will be evaluated.

### Item Generation and Content Development

Initial item development will be informed by the theoretical framework and the structured literature review described in this protocol, as well as by the research team’s clinical and methodological expertise within gerontological care [52]. In addition, qualitative findings from phase 1 will contribute empirical grounding through a structured process in which meaning units are identified, clustered into conceptual domains, and translated into candidate items.

While older adults’ lived experiences provide the empirical foundation for item content, item generation will not rely solely on participants’ verbatim expressions. In line with the notion of epistemic dialogue in patient-centered measurement [52], experiential knowledge will be treated as a vital but not self-sufficient source of evidence. Participants’ perspectives will inform item content and relevance, yet they will also be examined, conceptually refined, and integrated with theoretical frameworks (eg, person-centered care and lifeworld theory) and psychometric principles. In this way, the research team plays an active role in ensuring that items reflect both lived experience and a coherent construct suitable for rigorous measurement.

For each domain, an initial item pool (approximately 3‐5 items per domain) will be generated to capture varying levels of endorsement along the hypothesized continuum from lower to higher expressions of playfulness. Items will be formulated in Swedish using clear, concrete, and contextually appropriate language.

Item wording and content will be reviewed in 3 stages:

1. Internal expert review focusing on conceptual coherence and redundancy
2. Cognitive interviews with older adults to assess clarity, relevance, and interpretability
3. Iterative refinement based on empirical feedback and measurement considerations

All decisions regarding item inclusion, modification, or exclusion will be documented to ensure transparency.

### Documentation of Item Decisions

All stages of item development will be documented to ensure transparency and reproducibility. A structured decision log will be maintained throughout the development process, recording the origin of each item (eg, qualitative finding, theoretical refinement, or expert suggestion), revisions to wording, and the rationale for retention, modification, or exclusion.

During pilot testing and initial CTT analyses, the item pool will be retained in its entirety to preserve conceptual breadth. Early item removal will be avoided unless an item demonstrates clear redundancy or fundamental comprehension problems.

Subsequent Rasch analysis will provide a more rigorous evaluation of dimensionality, threshold functioning, item fit, and DIF. Decisions regarding item removal will be based on an integrated assessment of statistical performance and conceptual relevance. Items will not be removed solely on the basis of misfit statistics; instead, conceptual coverage, theoretical coherence, and construct validity will be weighed alongside empirical indicators.

This staged approach ensures that item reduction, if necessary, occurs only after a comprehensive psychometric evaluation and is supported by both measurement and conceptual considerations. The full decision process will be documented to provide a transparent audit trail linking qualitative findings, theoretical assumptions, and psychometric analyses to the final item selection.

### Prespecified Psychometric Criteria

Prespecified psychometric criteria have been defined to guide the evaluation and scale refinement of PLAY-SE. These criteria, grounded in established methodological literature, specify statistical thresholds and decision rules under both CTT and RMT (Table 2).

**Table 2.** Prespecified criteria for psychometric evaluation and scale refinement of the Play and Supportive Environments instrument.

Measurement property	Method	Criterion	Decision rule	Reference
Content validity	I-CVI^a^	≥0.78	Item revised if <0.78	[53]
Content validity	S-CVI^b^	≥0.90	Overall revision if <0.90	[53]
Data quality	Missing data per item	<10%	Investigate content, wording, and administration if ≥10%	[47]
Floor/ceiling effects	% lowest/highest category	≤20%	Consider revision if >20%	[47]
Internal consistency (CTT)^c^	Cronbach α	≥0.70 (group level)	Review scale if <0.70	[47]
Item-total correlation (CTT)	Corrected item-total	≥0.30	Review if <0.30	[47]
Dimensionality (CTT)	EFA loadings	≥0.40 on intended factor	Review cross-loading items	[38]
Rasch model fit (RMT)^d^	Item fit residual	−2.5 to +2.5	Review outside range	[54]
Threshold ordering (RMT)	Category probability curves	Ordered thresholds	Consider collapsing categories if disordered	[51,54]
Local independence (RMT)	Residual correlations (Yen Q3)^e^	<0.20 above average	Consider subtests or revision	[54,55]
Reliability (RMT)	PSI^f^	≥0.70	Review if <0.70	[54]
DIF^g^ (RMT)	2-way ANOVA on residuals	Nonsignificant after Bonferroni adjustment^h^	If significant, consider item split	[54,56]
Unidimensionality (RMT)	PCA^i^ of residuals + *t* tests	≤5% significant	If >5%, explore substructure	[51,57,58]
Item removal principle	Integrated conceptual + statistical evaluation	Not solely data driven	Remove only after Rasch and conceptual review	[36,52]

a I-CVI: Item Content Validity Index.

b S-CVI: Scale Content Validity Index.

c CTT: classical test theory.

d RMT: Rasch measurement theory.

e Yen Q3: residual correlation index used to assess local dependence in Rasch analysis.

f PSI: Person Separation Index.

g DIF: differential item functioning.

h Bonferroni adjustment refers to correction for multiple testing by dividing the significance level by the number of comparisons.

i principal component analysis

### Handling of Missing Data

Given the advanced age and varying functional capacity of participants, some degree of missing data is anticipated. The extent and pattern of missing responses will be examined descriptively. If item-level missingness exceeds 10%, this will be analyzed to determine whether it reflects systematic difficulties (eg, item wording, administration mode, or functional limitations). In CTT analyses, cases with excessive missing responses may be excluded from specific analyses. In RMT, the model accommodates some degree of missing data under the assumption of conditional independence; however, patterns of missingness will be evaluated to ensure that they do not reflect systematic bias.

### Ethical Considerations

The PLAY-SE program is conducted in accordance with the ethical principles for research involving human subjects, as outlined by the Swedish Ethical Review Authority. Ethical considerations are made throughout the research program, in accordance with the Swedish Ethical Review Act [59] and the Declaration of Helsinki [60]. Ethical approval has been obtained for the qualitative work in phase 1 (registration number 2025-00211-01). A complementary application will be submitted before data collection for later milestones. All data will be handled in accordance with established ethical standards for privacy and confidentiality. No identifying information (eg, names, initials, or other personal identifiers) will be included in the manuscript or supplementary materials. No compensation will be offered to participants.

Participation is voluntary and based on informed consent. Only older adults capable of providing informed consent will be included, and participants may withdraw at any time without consequence. As participants are older adults receiving municipal care, they may constitute a potentially vulnerable group due to age-related functional limitations and dependency structures.

To minimize risks and safeguard voluntariness, several measures are implemented. Recruitment is facilitated by designated care staff to identify eligible individuals; however, study information, eligibility confirmation, and informed consent are handled directly by the research team to emphasize independence from care provision. Participants are explicitly informed that participation or nonparticipation will not influence their care in any way.

Participants choose the time and location of interviews to ensure a familiar and secure setting. Focus groups are conducted separately for managers and staff to reduce power imbalances. During questionnaire administration, staff will not be present unless necessary for practical support. Any assistance provided follows a standardized protocol limited to reading items aloud and recording responses without interpretation. Given the risk of social desirability bias in care contexts, mode of administration (independent vs assisted completion) will be documented and examined analytically through DIF testing.

As discussions about playfulness may evoke emotional responses, participants are offered follow-up contact if needed. Confidentiality is emphasized throughout, and all research data are pseudonymized or anonymized as appropriate, securely stored, and managed in accordance with applicable data protection regulations and institutional policies.

A reference group of older adults has been involved from the outset and remains engaged throughout the research process. This participatory approach supports the ethical commitment to treating participants as situated knowers and strengthens the contextual sensitivity of instrument development.

Findings from the PLAY-SE program will be disseminated through publication in peer-reviewed journals and presentation at national and international conferences. Summaries will be prepared for practitioners and older adults and shared with participating care units and municipal stakeholders to facilitate knowledge translation into practice.

## Results

Ethical approval for the qualitative component of phase 1 was granted by the Swedish Ethical Review Authority (2025-00211-01; decision date: February 3, 2025). Data collection for the first qualitative study was conducted between February and April 2025 and included 15 older adults aged 68 to 100 years residing in nursing homes. The phenomenological findings from phase 1.1 have been published in March 2026 in the *International Journal of Qualitative Studies on Health and Well-being* [61].

Two PhD students were recruited to the program in September 2024 and September 2025, respectively, and an expert group was established in autumn 2025. The PhD students are funded, for four years each, by Kristianstad University (from 2024) and Red Cross University College (from 2025). These structures support the ongoing conceptual and psychometric development of the PLAY-SE instrument.

A complementary ethical application will be submitted prior to subsequent milestones, including pilot testing and large-scale psychometric validation. Pilot testing of the first version of the PLAY-SE instrument (approximately 100 participants) is planned for autumn 2026. Large-scale validation (250‐500 participants), including analyses based on CTT and RMT, is planned for 2027 to 2029. The overall project period extends to 2030.

## Discussion

### Principal Contribution and Implications

This study protocol outlines a structured and theory-informed approach to conceptualizing and measuring playfulness among older adults receiving municipal care. By integrating qualitative exploration with systematic psychometric evaluation, the program seeks to address the lack of a context-sensitive instrument grounded in the lived experiences of older adults receiving municipal care.

A central contribution of the program lies in its sequential design. The qualitative studies in phase 1 provide an empirical foundation for item generation, ensuring that the conceptualization of playfulness is informed by older adults’ own narratives of being playful in everyday life. This integration of phenomenological inquiry and measurement development aligns with person-centered and lifeworld-oriented frameworks, in which individuals are understood as situated knowers whose experiences shape both meaning and practice. Grounding item development in qualitative findings is expected to strengthen content validity before proceeding to large-scale psychometric testing.

In contrast to existing measures of adult playfulness, which primarily assess trait-level dispositions, PLAY-SE is designed to capture both being playful and acting playfully within specific care contexts. The inclusion of environmental enablers and constraints reflects the theoretical assumption that playfulness in later life emerges in the interplay between personal orientation, bodily capacity, and contextual conditions. This contextual framing differentiates PLAY-SE from more general playfulness scales and may enhance its relevance for municipal care of older adults.

The protocol incorporates a staged psychometric evaluation plan. By applying both CTT and RMT, the study will examine dimensionality, reliability, targeting, and measurement invariance. Prespecified decision criteria for model fit, item retention, and DIF aim to enhance methodological transparency. Nevertheless, the ultimate adequacy of the instrument will depend on empirical performance in diverse samples.

Several methodological challenges must be acknowledged. Older adults receiving municipal care represent a heterogeneous population with varying cognitive and functional capacities. Ensuring accessibility while maintaining measurement precision requires standardized administration procedures and documentation of assistance during completion. Furthermore, as the instrument is initially developed within Swedish municipal contexts, its broader applicability will require subsequent cross-cultural validation.

If satisfactory measurement properties are established, PLAY-SE may support future research examining associations between playfulness, well-being, participation, and care quality. It may also inform intervention studies exploring playful engagement as a potential resource for existential well-being in later life. However, such applications remain contingent upon empirical validation.

In summary, this protocol describes a theoretically grounded and methodologically transparent pathway toward measuring playfulness in later life. By combining qualitative depth with psychometric rigor, it aims to support the development of a context-sensitive instrument for person-centered municipal elder care.

### Strengths and Limitations

The literature review was conducted using a structured approach supported by the research platform Elicit. Although this tool enables broad concept-based search across a large academic corpus, it does not replace systematic database searches conducted across multiple bibliographic databases. As the review was intended to provide a focused conceptual overview rather than a formal systematic review, it may not have captured all relevant studies. Future research could benefit from a more comprehensive systematic search strategy across databases such as PsycINFO, PubMed, and CINAHL to further strengthen the empirical foundation.

## Abbreviations

CTT: classical test theory
DIF: differential item functioning
OAP: Older Adult Playfulness
OLIW: Other-directed, Light-hearted, Intellectual, and Whimsical (Playfulness Scale)
PLAY-SE: Play and Supportive Environments
RLR: reflective lifeworld research
RMT: Rasch measurement theory
SMAP: Short Measure of Adult Playfulness

## References

[1] Proyer RT (2013). The well-being of playful adults: adult playfulness, subjective well-being, physical well-being, and the pursuit of enjoyable activities. Eur J Humour Res.

[2] Proyer RT (2014). Playfulness over the lifespan and its relation to happiness: results from an online survey. Z Gerontol Geriatr.

[3] Brauer K, Stumpf HSC, Proyer RT (2024). Playfulness in middle- and older age: testing associations with life satisfaction, character strengths, and flourishing. Aging Ment Health.

[4] Dillon P, Gan DRY, Trivic Z (2022). Playfulness for brain health in mid- and late-life: scoping review to support preventive non-pharmacological interventions in community settings. Arch Phys Med Rehabil.

[5] Golland Y, Ben-David BM, Mather M, Keisari S (2025). Playful brains: a possible neurobiological pathway to cognitive health in aging. Front Hum Neurosci.

[6] Lubbers K, Cadwallader J, Lin Q, Clifford C, Frazier LD (2023). Adult play and playfulness: a qualitative exploration of its meanings and importance. J Play Adulthood.

[7] McCormack B, McCance T (2017). Person-Centred Practice in Nursing and Health Care: Theory and Practice.

[8] Burr B, Atkins L, Bertram AG, Sears K, McGinnis AN (2019). “If you stop playing you get old”: investigating reflections of play in older adults. Educ Gerontol.

[9] Hoppes S, Wilcox T, Graham G (2001). Meanings of play for older adults. Phys Occup Ther Geriatr.

[10] Merleau-Ponty M (2002). Phenomenology of Perception.

[11] (2024). Home is somewhere else: executive summary. https://mrinstitutet.se/download/18.12836cee194d834b690518e4/1740551595856/report-Home-is-somewhere-else_Executive-summary-2024.pdf.

[12] Neves BB, Sanders A, Kokanović R (2019). “It’s the worst bloody feeling in the world”: experiences of loneliness and social isolation among older people living in care homes. J Aging Stud.

[13] Gebhard D, Frank JI (2024). Everyday life and boredom of people living with dementia in residential long-term care: a merged methods study. BMC Geriatr.

[14] (2025). Socialtjänstlag (2025:400) [Swedish Social Services Act]. Swedish Code of Statutes.

[15] Proyer RT, Gander F, Bertenshaw EJ, Brauer K (2018). The positive relationships of playfulness with indicators of health, activity, and physical fitness. Front Psychol.

[16] Van Vleet M, Feeney BC (2015). Play behavior and playfulness in adulthood. Soc Pers Psychol Compass.

[17] Waldman Levi A, Bar-Haim Erez A, Katz N (2015). Healthy aging is reflected in well-being, participation, playfulness, and cognitive-emotional functioning. Healthy Aging Res.

[18] Elicit. https://elicit.com.

[19] Shen X (2020). Constructing an interactionist framework for playfulness research: adding psychological situations and playful states. J Leis Res.

[20] Schutter B, Vanden Abeele V Meaningful play in elderly life. https://www.researchgate.net/publication/242691512_Meaningful_Play_in_Elderly_Life.

[21] De Schutter B, Vanden Abeele V Designing meaningful play within the psycho-social context of older adults.

[22] Sayago S, Rosales A, Righi V, Ferreira SM, Coleman GW, Blat J (2016). On the conceptualization, design, and evaluation of appealing, meaningful, and playable digital games for older people. Games Cult.

[23] Parker C, Kennedy-Behr A, Wright S, Brown T (2023). Does the self-reported playfulness of older adults influence their wellbeing? An exploratory study. Scand J Occup Ther.

[24] Clifford C, Paulk E, Lin Q, Cadwallader J, Lubbers K, Frazier LD (2022). Relationships among adult playfulness, stress, and coping during the COVID-19 pandemic. Curr Psychol.

[25] Proyer RT (2012). Development and initial assessment of a short measure for adult playfulness: the SMAP. Pers Individ Dif.

[26] Proyer RT (2017). A new structural model for the study of adult playfulness: assessment and exploration of an understudied individual differences variable. Pers Individ Dif.

[27] Yarnal C, Qian X (2011). Older-adult playfulness: an innovative construct and measurement for healthy aging research. Am J Play.

[28] Loos E, Zhou J, Salvendy G Exergaming: meaningful play for older adults.

[29] Ricœur P (1992). Oneself as Another.

[30] Aho K (2020). Existentialism: An Introduction.

[31] Dahlberg K, Segesten K (2010). Hälsa Och Vårdande: I Teori Och Praxis [Book in Swedish].

[32] Dahlberg K, Dahlberg H, Nyström M (2008). Reflective Lifeworld Research.

[33] Gadamer HG (2013). Truth and Method.

[34] Husserl E (1970). The Crisis of European Sciences and Transcendental Phenomenology: An Introduction to Phenomenological Philosophy.

[35] Boateng GO, Neilands TB, Frongillo EA, Melgar-Quiñonez HR, Young SL (2018). Best practices for developing and validating scales for health, social, and behavioral research: a primer. Front Public Health.

[36] Wilson M (2023). Constructing Measures: An Item Response Modeling Approach.

[37] Nunnally JC, Bernstein IH (1994). Psychometric Theory.

[38] Streiner DL, Norman GR (2008). Health Measurement Scales: A Practical Guide to Their Development and Use.

[39] Stenner AJ, Stone MH, Fisher WP (2018). The unreasonable effectiveness of theory based instrument calibration in the natural sciences: what can the social sciences learn?. J Phys: Conf Ser.

[40] Petrillo J, Cano SJ, McLeod LD, Coon CD (2015). Using classical test theory, item response theory, and Rasch measurement theory to evaluate patient-reported outcome measures: a comparison of worked examples. Value Health.

[41] Andrich D (1988). Rasch Models for Measurement.

[42] Mari L, Wilson M (2014). An introduction to the Rasch measurement approach for metrologists. Measurement (Lond).

[43] Pendrill L (2014). Man as a measurement instrument. NCSLI Measure.

[44] Westergren A, Ahlström G, Persson M, Behm L (2021). Next of kin participation in the care of older persons in nursing homes: a pre-post non-randomised educational evaluation, using within-group and individual person-level comparisons. PLoS One.

[45] Huber E, Kleinknecht-Dolf M, Müller M, Kugler C, Spirig R (2017). Mixed‐method research protocol: defining and operationalizing patient‐related complexity of nursing care in acute care hospitals. J Adv Nurs.

[46] Schwartz‑Barcott D, Kim HS, Rodgers BL, Knafl KA (2000). Concept Development in Nursing: Foundations, Techniques, and Applications.

[47] Hobart JC, Riazi A, Lamping DL, Fitzpatrick R, Thompson AJ (2004). Improving the evaluation of therapeutic interventions in multiple sclerosis: development of a patient-based measure of outcome. Health Technol Assess.

[48] Hagell P, Westergren A (2016). Sample size and statistical conclusions from tests of fit to the Rasch model according to the Rasch unidimensional measurement model (RUMM) program in health outcome measurement. J Appl Meas.

[49] Tesio L, Caronni A, Simone A, Kumbhare D, Scarano S (2024). Interpreting results from Rasch analysis 2. Advanced model applications and the data-model fit assessment. Disabil Rehabil.

[50] Tesio L, Caronni A, Kumbhare D, Scarano S (2024). Interpreting results from Rasch analysis 1. The “most likely” measures coming from the model. Disabil Rehabil.

[51] Tennant A, Küçükdeveci AA (2023). Application of the Rasch measurement model in rehabilitation research and practice: early developments, current practice, and future challenges. Front Rehabil Sci.

[52] McClimans LM (2024). Patient-Centered Measurement: Ethics, Epistemology, and Dialogue in Contemporary Medicine.

[53] Polit DF, Beck CT (2021). Nursing Research: Generating and Assessing Evidence for Nursing Practice.

[54] Andrich D, Marais I (2019). A Course in Rasch Measurement Theory: Measuring in the Educational, Social and Health Sciences.

[55] Christensen KB, Makransky G, Horton M (2017). Critical values for Yen’s Q3: identification of local dependence in the Rasch model using residual correlations. Appl Psychol Meas.

[56] Hagquist C, Andrich D (2017). Recent advances in analysis of differential item functioning in health research using the Rasch model. Health Qual Life Outcomes.

[57] Tennant A, Conaghan PG (2007). The rasch measurement model in rheumatology: what is it and why use it? When should it be applied, and what should one look for in a Rasch paper?. Arthritis Rheum.

[58] Hagell P (2014). Testing rating scale unidimensionality using the principal component analysis (PCA)/*t*-test protocol with the Rasch model: the primacy of theory over statistics. OJS.

[59] (2004). Lagrummet.

[60] (2024). WMA Declaration of Helsinki - ethical principles for medical research involving human participants. World Medical Association.

[61] Bergman A, Haak M, Örmon K, Nivestam A, Westergren A, Nilsson Lindström P (2026). Older persons’ lived experiences of being playful in nursing home settings - a phenomenological reflective lifeworld research study. Int J Qual Stud Health Well-being.

